# Visit‐to‐visit blood pressure variability in mild cognitive impairment: A possible marker of Alzheimer's disease in the SPRINT study?

**DOI:** 10.1111/jch.14388

**Published:** 2021-11-21

**Authors:** Michiaki Nagai, Masaya Kato, Keigo Dote

**Affiliations:** ^1^ Department of Cardiology Hiroshima City Asa Hospital Hiroshima Japan

**Keywords:** Alzheimer's disease, insular cortex, mild cognitive impairment, vascular dementia, visit‐to‐visit blood pressure variability

The paper by de Havenon and colleagues^1^ in this issue of the Journal provides several new insights into the relationship between visit‐to‐visit blood pressure (BP) variability (VVV) and an increased dementia risk that was observed in a post‐hoc analysis of the Systolic Blood Pressure Intervention Trial‐Memory and Cognition in Decreased Hypertension (SPRINT MIND) cohort study of 516 hypertensive individuals with mild cognitive impairment (MCI). During the mean follow‐up of 2.6 years, the highest quartile of standard deviation (SD) in systolic BP (SBP) had a significant adjusted hazard ratio for dementia of 2.73 (95% CI: 1.31–5.69) independently of the average SBP. A similar significant relationship was also observed between the SD in diastolic BP and the incidence of dementia.^1^

One major cause of death and disability is vascular disease of the brain.^2^ While hypertension is associated with white matter lesions (which are a cause of vascular dementia [VaD]), a relationship between hypertension and Alzheimer's disease (AD) has also been described.^3^, ^4^ In fact, white matter lesions have been well known to pathologists since Alois Alzheimer first described AD in 1906. However, whether white matter alteration is a cause of the neurodegenerative disease was not established at that time.^5^

Many studies have indicated that VVV is associated with cognitive impairment^6^ and poses a risk of the development of dementia.^7^ Cerebral small‐vessel disease was suggested to be a pivotal pathophysiology.^8^

MCI is a well‐known syndrome that is thought to constitute a transition phase between healthy cognitive aging and dementia. The neuropsychological profile allows mainly two subtypes of MCI to be distinguished. One is amnestic‐type MCI, which may progress preferentially to AD, and the other is multiple domain‐type MCI, which may progress to AD and also to VaD, or may even represent a cognitive aging process qualified as normal.^4^, ^9^ Although the dementia subtype is not mentioned in the paper by de Havenon and colleagues,^1^ the patients with MCI in the analysis of the Alzheimer's Disease Neuroimaging Initiative (ADNI) progressed to AD within 12 months at a rate of 16.5% per year.^9^

There is a growing awareness that VaD and AD share similar pathophysiology in relation to VVV.^10^, ^11^, ^12^ Endothelial dysfunction, blood‐brain barrier disruption, and neurovascular unit dysfunction have been suggested as initial pathogenetic features in both VaD and AD, which could provide a link between arterial remodeling and AD.^4^

The causes of abnormal VVV are still being debated. Baroreceptor sensitivity (BRS) is another major determinant of BP variability.^13^ Increased large‐arterial stiffness contributes to BRS depression in hypertensives and has an important role in the increased BP variability in response to changes in the cardiac stroke volume.^14^ In contrast, VVV was shown to be associated with carotid artery remodeling.^15^ Increased VVV and arterial remodeling would thus provide a vicious circle via depressed BRS. Because increased arterial stiffness^16^ and decreased BRS^17^ were shown to be reduced in patients with AD, it is possible that the association between VVV and cognitive decline is moderated by arterial remodeling in the MCI‐to‐AD conversion.^10^, ^18^

Increased sympathetic nervous system activity is suggested to be involved in the pathophysiology.^19^ Increased hypothalamo‐pituitary‐adrenal axis activity might be associated with a sympathetic overdrive caused by factors such as emotional stress, environmental stress, and sleep dysregulation.^20^ Insomnia and long sleep duration, which were suggested to increase sympathetic nervous system activity, have been shown to have a relationship with increased VVV.^21^, ^22^ The low‐frequency/high‐frequency ratio was significantly higher in AD patients compared to controls, suggesting that patients with AD manifested a predominantly higher sympathetic nervous system tone.^23^ Increased sympathetic nervous system activity might thus be a pivotal factor in the relationship between VVV and the MCI‐to‐AD conversion.^10^

Cerebral hypoperfusion due to arterial remodeling enhances the production of the β‐amyloid peptide (Aβ). Arterial stiffening as well as microvascular dysfunction impair Aβ clearance and elevate brain Aβ levels.^24^ Increased large arterial stiffness might exert a direct effect on cerebral penetrating arteries, associated with an altered structure and function. This process subsequently has a harmful effect on perivascular Aβ from the brain to the perivascular space via the cerebrospinal fluid drainage. A disruption of vascular dynamics and reduced perivascular flow of Aβ thus causes decreased Aβ clearance.^24^ As a consequence, arterial remodeling has a relationship with cerebral Aβ deposition.

Recent studies have supported the notions that (1) the cardiovascular system is regulated by a cortical network consisting of the insular cortex (Ic), anterior cingulate gyrus, and amygdala, and (2) this network regulates the central autonomic nervous system in relation to emotional stress.^25^ Because the Ic is located in the region of the middle cerebral arteries, its structure tends to carry a higher risk of cerebrovascular disease.^25^ In clinical studies, Ic damage has been associated with increased BP variability, depressed BRS, and higher plasma levels of catecholamine.^25^, ^26^

AD is associated with both Ic pathology and autonomic dysfunction.^27^, ^28^ Braak and Braak demonstrated that neurofibrillary tangles (NFTs) progress through the brain in a highly structured sequence that begins in the mesiotemporal cortex and progressively invades the association cortex in the frontal, temporal, and parietal lobes.^29^ AD is merely the final stage of a pathological process that spans decades.

Earlier studies demonstrated a hierarchical sequence of AD pathology that includes the Ic.^27^, ^28^ This may explain why AD has effects on BP via central autonomic regulatory functions. Brainstem nuclei are affected too late in the Braak sequence to explain preclinical dysautonomic symptoms, and AD pathology in the Ic is a more likely origin of autonomic dyscontrol in the early stage of AD.^27^, ^28^ The Ic is affected at a preclinical stage in the Braak sequence (ie, stage III of six). AD pathology reaches the Ic at a ‘preclinical’ stage before ‘dementia’ can be diagnosed.^27^, ^28^ It is hypothesized that autonomic dyscontrol (which is commonly observed in non‐demented elderly individuals without significant cardiovascular disease) reflects subclinical stages of AD pathology affecting the Ic.^27^, ^28^ In fact, a significant relationship between the Braak sequence and higher VVV was recently reported.^30^ Regarding the central autonomic nervous system, NFT deposition in the Ic would increase VVV.^19^ These factors might also be associated with the MCI‐to‐AD conversion. Figure 1 illustrates the possible pathophysiology of VVV for both VaD and AD in relation to Aβ and NFT deposition.

**FIGURE 1:**
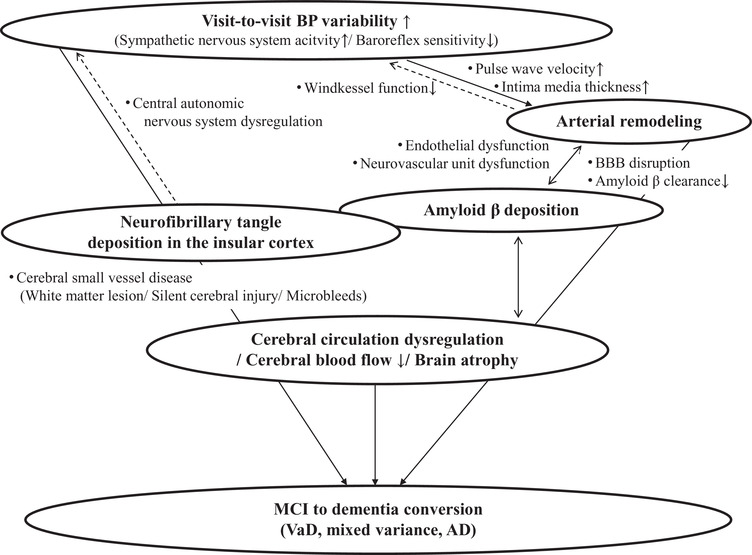
The pathophysiology of visit‐to‐visit blood pressure (BP) variability (VVV) underlying the conversion from mild cognitive impairment (MCI) to Alzheimer's disease (AD). The relationship between VVV and artery remodeling suggests that VVV is a risk factor for the development of MCI, vascular dementia (VaD), and AD. The *continuous‐line arrow* shows known relationships: the *solid‐line arrows* indicate possible relationships. Reconstructed from Nagai and colleagues^10^

From this standpoint, arterial remodeling as well as AD pathology might serve as potential moderators of the relationship between increased VVV and cognitive decline in MCI. The data presented in the paper by de Havenon and colleagues^1^ will have a greater impact if the mechanism underlying that relationship, specifically the MCI‐to‐AD conversion, is described.

## CONFLICT OF INTEREST

The authors declare no conflict of interest.

## AUTHOR CONTRIBUTIONS

Masaya Kato, Michiaki Nagai, and Keigo Dote contributed to the conception of the study. Masaya Kato and Michiaki Nagai to the literature analysis and manuscript preparation. Michiaki Nagai wrote the manuscript. Masaya Kato, Michiaki Nagai, and Keigo Dote contributed to the design and revision of the figure. All authors have read and approved the manuscript.
